# Perspectives of People with a Chronic Disease on Participating in Work: A Focus Group Study

**DOI:** 10.1007/s10926-016-9694-6

**Published:** 2017-01-18

**Authors:** Marloes Vooijs, Monique C. J. Leensen, Jan L. Hoving, Haije Wind, Monique H. W. Frings-Dresen

**Affiliations:** 1 Coronel Institute of Occupational Health, Academic Medical Center, University of Amsterdam, P.O. Box 22700, 1100 DE Amsterdam, The Netherlands; 2 Research Institution for Insurance Medicine, Amsterdam, The Netherlands

**Keywords:** Chronic disease, Occupational health, Employment, Solutions, Social support

## Abstract

*Purpose* To explore solutions that people with a chronic disease use to overcome difficulties they experience regarding participating in work, and the support they require to identify or implement these solutions. *Methods* Focus groups were held to explore solutions and support requirements of people with a chronic disease. Participants were recruited through a research institution’s patient panel, a patient federation and personal networks. Analysis was conducted by means of open and selective coding, using the MAXQDA software package. *Results* Five focus groups were held with 19 participants with different chronic diseases. Solutions that were identified included learning to accept and cope with the disease, which is frequently supported by family and friends. Disclosing the disease to employers and colleagues, identifying active ways to help with duties, and implementing adaptations to the work environment were all effective solutions with the help, empathy and understanding of people in the work environment. Solutions mostly supported by patient associations included providing sufficient information about the disease, relevant help and protective legal regulations regarding work participation. Finally, health professionals could support solutions such as incorporating periods of rest, promoting self-efficacy and gaining insight into an individual’s ability to participate in work. *Conclusions* People with a chronic disease suggested various solutions that can help overcome difficulties surrounding participating in work. Support from friends and family, patient associations, employers, colleagues and occupational health professionals is needed to help identify and implement suitable solutions.

## Introduction

A substantial number of people are affected by a chronic disease [1], with 28% of working-age people having a chronic disease [2]. According to the World Health Organization, a chronic disease is defined by a long duration and a generally slow progression [3]. A chronic disease can negatively affect work participation because of experienced limitations through the disease [4–7]. Consequently, many people with a chronic disease work fewer hours or are not employed at all [8, 9].

There is an increasing focus on the individuals’ self-management of the disease and its effects [10–14]. In addition, in the Netherlands, people with a chronic disease have a shared responsibility regarding participation in work [15], in which they are expected to work with occupational health professionals to determine a plan of action to overcome the difficulties they experience [15]. In this plan of action, steps and solutions are established, in which both employer and employee are responsible for the execution of these steps to improve work participation of the individual [15].

Previous research shows that research on experiences of people with a chronic disease mainly focuses on the experienced limitations and difficulties [5–7, 16, 17]. This study adds by focusing on the solutions that are used by people with a chronic disease to overcome these difficulties. Earlier research regarding solutions only reported on work accommodations [18, 19]. We aimed to extent this knowledge and to gather information on other solutions as well. Because earlier research found that many aspects are not related to a specific diagnoses [20, 21] we aimed to find solutions, irrespective of diagnosis.

Although people with a chronic disease share responsibility in their process of participation in work, some situations challenge people to develop and implement solutions, in which support from others is needed. A pre-requisite for providing effective support to enable people with a chronic disease to participate in work, is to have a good understanding of their needs. This includes learning what type of solutions are used by people with a chronic disease, what type of support is required and who needs to provide the support. Therefore, this study focuses on obtaining information on the following research questions: What solutions do people with a chronic disease use in order to facilitate and manage their participation in work? And what support do they need in this regard?

## Method

For this study we used a qualitative approach, to explore the perspectives of people with a chronic disease with respect to their participation in work. Since work plays an important role in the lives of people with different diagnosis and both with or without employment, we aimed to gather information on solutions used by people irrespective of their diagnosis and work status. We ran focus groups to help individuals -by gaining greater awareness of solutions others use- to become aware of the range of solutions they (un)consciously use themselves to participate in work. Items of the consolidated criteria for reported qualitative research (COREQ) [22] were used in order to improve the design and quality of reporting qualitative research. The Medical Ethics Committee of the Academic Medical Center determined that no ethical approval was required for this study (trial number: W15_174 # 15.0211).

### Participants

We recruited participants for this study by sending invitations to members of a patient panel of the NIVEL research institution and to a large patient association (Ieder(In)). The members of both the patient panel and the association included people with a range of different chronic diseases, which they generally suffered from over a long period of time. In addition, members of both the patient panel and association had a relation with work, meaning that they either had employment or that they wanted or needed to return to work. Participants were also approached via a standardized notice posted on the social media sites of various patient associations. Lastly, we recruited participants through the researchers’ personal networks.

Those people who indicated interest in participating received an information leaflet and were invited to send an email to one of the research team members (MV), providing their gender, age, chronic disease, work status and contact details. The researcher (MV) then contacted the applicants by telephone to provide additional information about the study and to explain the sampling procedure. The sampling strategy aimed to include an equal division of gender, age and work status and the inclusion of people with different diagnosis in each focus group. People were eligible to participate if they suffered from a chronic disease, were aged between 18 and 65 years and were either in employment or seeking employment. We defined a number of specific categories of chronic diseases and included no more than three people from each in our overall sample in order to achieve an equal representation of various chronic diseases. We then scheduled focus groups with between four and six participants. Informed consent was obtained from all participants included in this study.

### Data Collection

The focus groups were held between October 2015 and December 2015, at the Academic Medical Center (AMC) in Amsterdam, the Netherlands. The duration of the focus groups was a maximum of 2 hours. The groups were run by a moderator and an observer, both working in the field of occupational health. ML, a female researcher, moderated the discussion in four focus groups and HW, a male researcher, moderated the discussion in one focus group. The observer (MV), a female researcher, took notes on non-verbal communication, group dynamics and the topics covered during the focus groups on a standardized form. At the start of each meeting, the moderator explained to the participants the purpose of the study and the role of the moderator and observer. In addition, participants were informed that all information obtained prior to or during the study would be handled confidentially and that an audio-recording would be made of the groups’ discussions. In our aim to provide insight in the role of the individual with a chronic disease in their work participation, we formulated the questions: What solutions do you currently use or have you used in the past to overcome difficulties you face in relation to work participation? What support do you currently need or have you needed in the past to identify or implement those solutions? Who do you need or have you needed support from and in what form? No other people were present during the focus groups besides the participants and the researchers.

### Data Analysis

The recordings of the focus groups were transcribed verbatim. The transcripts were coded using the MAXQDA software program (Verbi GmbH, Marburg, Germany), applying open and axial coding [23]. First, two researchers (MV and ML) coded one of the focus group transcripts independently using open coding [23], after which they discussed the codes until they reached a consensus. The first researcher (MV) then coded the remaining four transcripts using open coding, followed by an additional check on one of these transcripts conducted by the second researcher (ML). Thereafter, the retrieved open codes were categorized in themes [23], which are described in the results. During the process the list of open and axial codes was repeatedly discussed by the entire team to check the codes and to establish consensus.

## Results

As previously stated, we originally scheduled focus groups containing four to six participants. However, due to last minute cancelations, some focus groups contained only three participants. We initially conducted four focus groups, after which we decided to conduct another focus group meeting by means of a final check of data saturation. The fifth focus group yielded results in line with our previous findings; we therefore decided that data saturation had been achieved.

We conducted a total of five focus groups, each including three to six participants having different chronic diseases, such as: whiplash, kidney disease, rheumatic arthritis, osteogenesis imperfecta, visual handicap, dysmelia, Lyme disease, thrombophilia, repetitive strain injury, diabetes, cancer, and dystrophy. The total sample contained 19 participants; 10 women and 9 men. The mean age of the participants was 50 (SD: 10.7; range 28–62) years old. A total of 15 people had employment, of which 11 worked in an organization and four people worked as a freelancer.

### Results Per Theme

Our findings are presented according to theme: the various solutions are set out first, followed by support required or received. Most of the results relate to those in employment, who shared their solutions for either retaining work or returning to work after a period of absence due to illness. Four of the participants were not currently employed, which we have specifically noted in our results. We included quoted statements made by participants during the discussions to illustrate our findings.

### Acceptance and Coping

The participants stated that having a chronic disease was difficult to accept. One solution reported was to actively work on accepting the disease and its effects. In terms of coping strategies, the participants recommended focusing on what they could still do rather than what they could no longer do due to the disease.

“But you need to focus on what you can do, rather than on what you can’t do. That is my motto in life.” [Participant 18]

Some participants mentioned that they had to ‘start from scratch’ to rebuild their personal and working lives. Others coped by ‘pushing through’ when they felt out of energy or when experiencing the limitations imposed by the disease. In contrast, one participant coped by concealing the chronic disease, so that others were not aware of it.

In terms of support, the participants indicated that they needed help learning to accept the disease, which they generally received from family and friends. Occupational health professionals also helped by acknowledging the disease. Participants found it valuable to receive guidance from a professional who had experience dealing with their specific disease. One participant with multiple morbidity needed a professional who could provide a useful overview of all the diseases concerned. The participants stated that it was important not to struggle with the disease alone.

### Insight into Abilities and Limitations

Participants stated that one solution for facilitating work participation was to gain insight into their own abilities and limitations in relation to work, which they had done by reaching the limits of their abilities on one or more occasions.

“It’s mainly about finding your own limits. The saying goes “once bitten, twice shy” – well ... I think I’ve been bitten at least ten times already ...” [Participant 11]

Participants would have liked to receive help identifying their abilities and limitations from others, e.g. employers, coaches, family members, occupational health professionals and other patients. One unemployed participant suggested that occupational health professionals could help by providing information about what kind of jobs could be performed by people with specific chronic diseases.

“After I stopped the chemo, I felt I was ready to go back to work again just like before. Well, it didn’t quite work out that way – and it took me far too long to realize it. And also if you are then applying for 40-hour jobs, you are certainly not on the right track. Someone should have pointed this out to me.” [Participant 17]

### Boundaries

Another solution, according to the participants, was learning to set boundaries for both themselves and others, to help prevent participants from exceeding their physical abilities and to manage expectations.

Many participants had difficulty maintaining their boundaries, but found ways to help them to do so, such as setting an alarm in order to avoid working too long. Others received help from their employer in this regard.

“Whenever I work too long, my supervisor says ‘Time to go home!’. Then she simply says: ‘Since you’ve worked half an hour more today, tomorrow you can leave half an hour earlier.’ So now she’s really the one who sets my limits.” [Participant 10]

Participants also indicated a need for skills training to learn to communicate their boundaries to others.

### Disclosure

A further solution reported was disclosing the chronic disease to employers and colleagues and making them aware of how it affects the participants’ work and work environment. Participants stated that opening up about their abilities and limitations as a result of their disease generated understanding from employers and colleagues.

“So, being open allows you to let other people see what you can or can’t manage and what your needs are.” [Participant 1]

One participant added that the explanation has to be given in simple and easily comprehensible terms to enable others to relate to it. Other participants noted that it helped to use humor in the explanation. Some unemployed participants stated that they preferred not to disclose their disease due to previous negative experiences. In contrast, however, one participant chose to tell a potential employer about the disease during the salary negotiations, which proved to be a positive experience. Disclosure also paved the way for setting boundaries, making relevant agreements and enabling help from colleagues.

With regard to support, participants expressed a need for empathy, interest and understanding from employers and colleagues after disclosing their disease.

### Obtaining Information

Unemployed participants put forward obtaining information about companies who hire people with chronic diseases or about the advantages of hiring employees with chronic diseases for employers as a solution.

“So, basically it would help me to know of any companies that say “We hire people like you”.” [Participant 12]

Participants indicated that they needed support to acquire information about the disease itself, the types of help available, possible adaptations and how to communicate with health professionals. They stated that they currently acquired this information from patients’ associations and hospital outpatient clinics. For those without employment, support was required to obtain information on regulations concerning work participation and organizations that are willing to hire people with a chronic disease. According to one unemployed participant, employers could also provide support by familiarizing themselves with the rules surrounding hiring people with a chronic disease.

### Self-efficacy

The participants listed various solutions that centered on the need to believe in their own qualities and to effectively communicate these qualities in order to participate in work. They observed that had they succeeded in obtaining or keeping their jobs by knowing their value to the organization and persuasively communicating this to their (potential) employer.

“Eight years ago, during a job interview, I said “I know perfectly well what I’m capable of. Once I’m hired, you’ll see that I’m an extremely good worker. Except that, in this situation, I also have certain limitations. Unfortunately, I can’t do any night work.” That’s essentially how I put it.” [Participant 16]

Participants indicated a need for courses to help gain insight into their strengths in order to become aware of what value they could hold for an organization.

### Skills Development

The participants reported that taking courses and skills training is a valuable solution. Unemployed participants also mentioned that training on social skills and job application skills would be useful.

“I’ve received training on applying for jobs where I learnt how to write application letters. And during those sessions, I also learnt to take things step-by-step, focusing on one step at a time: not expecting to immediately get the job, but first focusing on writing a good letter. And only after that starting to look forward to being called for an interview. Then to just see the interview as a chance to gain interview experience – this in itself is a positive thing – instead of immediately expecting to get the job.” [Participant 19]

In addition, another unemployed participant put forward staying ‘active’ by doing voluntary work as a means of developing and applying necessary skills.

Support that could be provided in this regard included receiving information about courses that are available, which is currently mostly provided by patients’ associations.

### Managing Energy Levels

Another solution introduced by participants was incorporating rest periods before and after work, in order to be able to effectively work the following day. One participant also incorporated rest periods during work by assigning certain tasks to his employees.

“But your body automatically starts operating at half-capacity. And as soon as you notice this, you have to accept that you are no longer capable of functioning at full capacity. Fortunately, in my line of work, this usually doesn’t mean getting less work done. It only means that people need to work more independently and show me the results. In this way, I can take a short nap and return after an hour to take a look at what they have done with the scenes.” [Participant 13]

A second participant incorporated rest periods in both the private and working life by learning to strive for smaller goals. A third participant hired an assistant for the household in order to save energy, to help maintain a balance between work and personal life. In contrast, other participants chose to put all of their energy into their work and simply deal with the consequences of this effort, such as resulting lack of energy or pain, at home. Work was their first priority and their personal life came second. One unemployed participant sought a job involving less strain in terms of duties and hours in order to sustain work in combination with managing their chronic disease. Another solution mentioned was inquiring whether it would be possible to work on a part-time basis instead of full-time.

Suggestions relating to support included a coach who could help participants learn how to manage their energy during the day and to identify tasks that could be assigned to others. The participants also expressed a need for skills training to learn to set smaller goals in order to maintain their ability to work.

### Asking for Help

Various participants advocated actively asking others for help, for example, help from colleagues to perform certain work tasks that they were no longer able to perform due to their disease. They also recommended trading particular work tasks with colleagues in order to be able to perform more suitable tasks in view of their disease.

“Can you take care of the printing work for a bit, while I take over some of your tasks during that time?” Simply keep negotiating over the tasks to be done and, with a bit of help from your colleagues, you can manage.” [Participant 4]

Employed participants enabled help regarding necessary adaptations in their work or to their work environment. Unemployed participants reported asking help from occupational health professionals in order to deal with problems regarding the rules governing or payment of their disability benefit now or in the near future. Some asked for help by spreading the word that they were searching for employment so that others could help find vacancies. One participant arranged the reintegration into work by actively approaching others for help.

The participants advised that here they needed information from occupational health professionals on what forms of support are available. Employers could provide support by responding to the requests of employees with a chronic disease for help. One participant indicated that skills training on asking for help would be valuable.

### Mutual Agreements

Making clear agreements with employers about work duties, hours, location and doctor or hospital appointments during work hours, and communicating these agreements to colleagues was put forward as an additional solution. This helped participants to manage expectations and consequently they received fewer negative comments from their colleagues.

“During my performance appraisal interview, I agreed with my supervisor to make Friday my regular day off and to work at home on Tuesdays. So now it’s down on paper and everyone knows about it.” [Participant 2]

Participants indicated a need for empathy and understanding on the part of both employers and colleagues so that they could feel that they had the opportunity to make and communicate such agreements. One participant found it helpful to have the occupational health professional set down details of the chronic disease in a report to take to the consultation with their employer. This made the disease ‘official’ for others.

Unemployed participants preferred to receive support in the form of mediation for both securing and retaining employment. They felt this could help them make agreements concerning hiring a person with a chronic disease and ensuring that the employee’s abilities would not be exceeded while working.

### Autonomy at Work

The participants commented on the importance of having the autonomy to work from home or schedule their own work tasks. This helped them take the limitations imposed by their chronic disease and doctor or hospital visits into account when planning their work.

“If a deadline is set for Friday, for me it automatically shifts to the preceding Tuesday or Wednesday, so that if I have a bad day, I can still finish it on the Wednesday or Thursday.” [Participant 3]

The participants noted that employers could provide support in this regard by allowing participants a degree of autonomy in their work.

### Adaptations to the Work Environment

A final solution the participants put forward was making appropriate adaptations the work environment, depending on their specific disease and needs.

They indicated that occupational health professionals and patient organizations could give support in the form of providing information about environmental adaptations and supportive devices that are available. Employers can also support participants by approving and financing supportive devices and workplace adaptations.

“My employer paid for a specially adapted chair for me.” [Participant 2]

### Types of Support from Health Professionals

With regard to solutions and self-management, various participants said that, in their experience, they themselves were responsible for how they dealt with the effects of their disease at work, but that they also needed support. The preferred type of support from occupational health professionals was to take a personal approach. In addition, one participant stated that professionals need to respond to the needs and requests of individual participants.

“I notice that there are many targeted solutions available, such as a specific course or a particular possibility. Whereas it should be the other way around – they should listen to you and ask you what you need, what kind of help you require. Asking the question is part of the solution.” [Participant 11]

In a similar vein, a second participant reported receiving unwanted support and agreed that the patient should be able to specify what kind of help is needed. A third participant wanted to be treated with trust instead of mistrust by occupational health professionals. The participants preferred proactive types of support in which professionals actively provide solutions to participants. They also said that professionals should be both objective towards and easily accessible to those in need of support. Many stated that professionals often focused on just one aspect of their life, e.g. their medical status or work situation, whereas participants needed them to consider the ‘big picture’ of the patient’s life.

The participants mentioned that they felt as if occupational health professionals were not adequately prepared for appointments and urged professionals to read participants’ files before meeting them. One noted that professionals do not need to know all of the patient’s symptoms, but should understand what complications their symptoms cause.

One participant said that employers also need to receive support, because they are not trained on how to deal with employees with a chronic disease. Another asserted that organizations can support people with a chronic disease by providing easy accessible professional support, such as a company nurse.

## Discussion

The purpose of this study was to explore the solutions that people with a chronic disease implement to facilitate their participation in work, and what support they need in finding or implementing solutions to overcome the various difficulties they experience. The participants reported a large number of solutions, either focused on themselves (e.g. accepting and coping with the disease, gaining insight into what they are now capable of, believing in themselves) or focused on their job and workplace (e.g. having a degree of autonomy at work, making adaptations to the workplace), for which they generally required support from their employer, colleagues and occupational health professionals.

Although focus groups were held with smaller groups sizes than intended, which may have had effect on group dynamics, we were able to gather various perspectives and to obtain data saturation. A strength of this study was the interaction amongst participants with various chronic diseases, in which we were able to gather information from all perspectives, irrespective of diagnosis. Through this interaction, participants were enabled to learn about other participants’ experiences and solutions, which increased their awareness of the range of solutions for participating in work. However, our decision to focus on solutions that people have already implemented, may mean that we missed out on the perspectives of those who have difficulty finding solutions to participate in work. It is also possible that the participants who responded to our invitation, experienced more difficulty in participating in work than other people with a chronic disease. This may indicate that not all people with a chronic disease require solutions in order to participate in work or do not require help identifying and implementing them.

The themes in our study are in line with themes of other studies concentrating on promoting or facilitating factors regarding work conditions or quality of working life for people with a chronic disease [8, 18, 19, 24]. One example is the importance of accepting the disease and learning to cope with it. This seemed as an important step – one that then facilitates the application of other solutions, particularly relating to work participation. Previous research agrees that accepting the disease enables people with a chronic disease to continue to work, provided that they know how to look after themselves in the work environment [24]. This implies that non-acceptance of a chronic disease can create barriers to participation in work. Our participants suggested that it should be among the tasks of occupational health professionals to support people with a chronic disease in learning to accept and cope with their disease.

With regard to disclosing their disease to others, some of our participants opted for disclosure while others preferred non-disclosure. In a positive sense, disclosure can certainly facilitate the implementation of other solutions, such as obtaining support and adaptations to the workplace [24], being able to communicate a patient’s capacity for work and setting realistic expectations [24, 25]. On the other hand, negative experiences regarding disclosing the disease led some participants to decide not to disclose their disease to their employer or colleagues. Research has also revealed that some people are afraid to disclose their disease [26], often due to stigmatization [27]. In summary, disclosing the disease may lead to more flexibility from participants’ work environments, provided that they have empathic and understanding employers and colleagues.

Practical solutions, such as making adaptations in the workplace, were frequently identified in our study, as well as in earlier research as a means of facilitating work participation [5, 18, 19, 24]. Our participants reported that next to workplace adaptations, also a high degree of autonomy provided a helpful solution. With regard to having autonomy, both our study as well as earlier research [18, 19] stated that facilitating people to work from home, allowing them to plan their own work, trading work tasks with colleagues and incorporating rest periods helped them to retain their jobs. This implies that if people are afforded the opportunity to match their working hours and location to their abilities and limitations at the time by employers and colleagues, they are more able to participate in work.

In contrast to practical solutions, previous research as well as our participants, indicated that they have experienced more problems due to lack of understanding from employers and colleagues [18]. Support was reported to be important because good support effectively promotes participation in work [5, 24, 25, 28], and was found to be necessary for people to obtain support in order to manage their disease and its effects themselves [29]. Our participants likewise reported that people themselves must allow and actively enable others to support them, so that they could get the support the needed.

The results of this study demonstrate that people with a chronic disease are capable of identifying effective solutions themselves and taking responsibility of their own participation in work. Based on these results, we urge occupational health professionals to involve people with a chronic disease more closely in finding solutions to their participation in work. This leads to, not only greater acceptance, but also higher compliance with professional advice [30]. In addition, based on the results of this study, health professionals need to provide personalized advice to individual patients, taking account of their specific situation and personal circumstances. With regard to employers, we recommend that they communicate with the individual what they need to return to work or to retain work to facilitate in these solutions to the level they are able to.

## Conclusion

This study reveals a number of solutions for overcoming difficulties in order to participate in work, from the perspective of people with a chronic disease. Various solutions are reported, either applying to the person itself or related to the work and work environment. Some require the help of others, in which family and friends, employers and colleagues, as well as health professionals can provide support to find and implement these solutions.
